# Re-examining provider perceptions of best pre-war practices: what elements can help opiate agonist therapy programs in Ukraine successfully survive the crisis?

**DOI:** 10.3389/fpubh.2023.1259488

**Published:** 2024-01-04

**Authors:** P. Dellamura, A. Meteliuk, T. Fomenko, J. Rozanova

**Affiliations:** ^1^Department of Internal Medicine, Section of Infectious Diseases, Yale University, New Haven, CT, United States; ^2^ICF – Alliance for Public Health, Kyiv, Ukraine; ^3^Ukrainian Alliance for Public Health, Kyiv, Ukraine

**Keywords:** Ukraine, crisis, war, opioid agonist therapy, OAT providers, addiction treatment, pre-war practices

## Abstract

**Purpose:**

This paper seeks to explore and understand what constitutes successful opioid agonist therapy (OAT) programs from the views of Ukrainian OAT providers in their context-specific accounts. Prior to the ongoing war the Ukrainian addiction treatment system made great strides to expand its OAT program and increase the number of patients. Since the beginning of the war there has been much alarm that those hard-earned successes will be destroyed. However, emerging evidence suggests that the Ukrainian OAT programs have shown signs of resilience in the face of adversity albeit at great cost. What aspects of the OAT programs developed prior to the crisis have been helpful to allow them to weather the storm? Using the data from 24 addiction treatment providers, this paper describes the essential elements of the OAT programs that preceded the current crisis which made them robust over time. By examining the narratives of the participants interviewed pre-war and pre-COVID-19 the paper reveals structural and cultural elements of the OAT programs before the perfect storm that are likely to endure. To the best of our knowledge, no one else has investigated OAT providers perspectives in Ukraine prior to the crisis which makes this paper extremely salient to understand both the robustness and the vulnerability of MAT programs in Ukraine during the war and going forward.

**Methodology:**

The data come from qualitative semi-structured interviews with 24 OAT providers throughout 5 regions of Ukraine. Participants included front-line clinicians, head narcologists, and chief doctors from TB clinics, district hospitals and drug addiction centers. Using a coding scheme of 103 inductively developed categories we explored participants’ perceptions of their OAT program.

**Findings:**

In the stories shared by clinicians pre crisis, three major interconnected themes focused on economic uncertainty at the institutional level (leading to under-staffing), structural capacity of the program, and clinicians’ professional identity, shaping differing views on application of rules for administrative discharge, take-home dosing, and the potential for scale-up. Knowing the data collection was completely unbiased to the current crisis, interpreting the findings helps us understand that OAT clinicians have had “years” of training under conditions of duress in Ukraine to overcome barriers, find creative solutions and form a support network that became indispensable in surviving the current humanitarian catastrophe.

**Conclusion:**

In the discussion we point out that the current crisis magnified the pre-existing challenges as the providers approach toward overcoming them was already largely present before the crisis (just on a different scale). The underlying fragility of resources was a constant since OAT inception in Ukraine. Historically, providers in Ukraine operated in a system that was under-funded in the absence of solid governmental funding for OAT programs, yet they came up with solutions which required ingenuity that they took pride in. This gives hope that addiction treatment in Ukraine and OAT programs will not be casualties of this humanitarian crisis and providers and their patients will persevere.

## Introduction

The Covid-19 pandemic shut-down coupled with the Russian invasion in Ukraine that began on February 24, 2022, constituted the sudden crisis, whose one-two punch impact was felt on many levels in Ukraine. One documented impact was the risk of abrupt interruption of medication and OAT service delivery to 17,232 people on OAT at the beginning of the invasion. Russia’s attack on Ukraine resulted in displacement of approximately one-third of the population. Displacement had an immediate impact on OAT patients who relied on local pharmacies and OAT programs for daily dosing (1). In a desperate attempt to minimize the abrupt interruption of medication, OAT providers sent Telegram-SMS messages to patients offering to meet them in designated areas to personally deliver take-home doses.

The documented reaction of the Ukrainian OAT services to the impact after Russia’s 2022 invasion was the relocation of OAT programs to the western region of Ukraine. OAT providers continued to share information with each other via Telegram-SMS to minimize the loss of patients and to continue care and medication assistance. In stark contrast to pre-crisis policy, OAT providers allowed take-home doses (10- to 30-day supply) as local pharmacies access to emergency medication was disrupted.

While inspiring to emergently look for solutions, the crisis exposes and amplifies pre-existing deficiencies as well as strengths. This paper aims to describe the status quo in the addiction treatment system in Ukraine and its relative strengths and weaknesses before the dual crisis hit. This is significant to unpack the characteristics of the addiction treatment system and the experiences of the addiction treatment providers pre-crisis to understand what issues pre-dated the crisis and with which the facilities and staff were already familiar and to which they have developed robust responses. Likewise, looking at the addiction treatment system pre-crisis when there was no major disruption can help us explain why certain challenges were subsequently bigger than others and what helped the facilities and the staff to survive.

To understand what was at the heart of the status quo prior to crisis will ultimately help to build a viable post-crisis strategy for addiction treatment in Ukraine and elsewhere. And in so doing, the point of view of providers is key, but coincidentally has been largely overlooked, making analysis of addiction treatment providers’ accounts in Ukraine using exclusive data obtained pre-crisis distinctive and extremely salient.

When we started researching this topic 4 years ago, we found only 3 publications in the literature focused on testimonies of Ukrainian addiction treatment providers themselves (2–4). Now there are only 5 publications (1, 5) signifying that all through COVID all the attention was placed on patients and service users, but little attention has been paid to this provider group and that is why this data is so significant. Considering how important it is, there was very little attention paid.

This article presents findings from the analysis of data collected in 2017 from OAT providers to understand barriers and facilitators of the success of the OAT system in Ukraine (and never yet published). The data was collected during the pre-crisis time and looking at findings from the vantage point of the present time sheds light on how these barriers underlie the subsequent risks and how facilitators helped to offset them especially during the war and humanitarian crisis.

This article highlights healthcare staff’s rarely heard perspective on the challenges that pre-dated crisis in the Ukrainian addiction treatment system. It reflects on whether having learned how to navigate these challenges over the years gave buttressing against the crisis to the addiction care providers, allowing them to lean on well-practiced skills. But where is the boundary? At what point does it break? What is the call-for-action from this knowledge? This article contributes to finding the answer to this question.

**Interestingly, before the crisis, addiction treatment providers drew their wish lists** including their recommendations that in their view would improve the addiction care system. These wish lists also revealed the fundamental limitations and deficiencies of the system that the crisis had laid bare. Although these recommendations are made in real-time, they existed pre-crisis but were ignored by the governmental officials and policymakers.

The problems pre-date the crisis and providers concerns must be taken seriously because it appears that what they have identified over the years are indeed the issues of the system. The wish list is like an “evidence-based time capsule.” Although now the call for action is not much different than what providers asked for years ago, it is now of greater significance, and the voices of addiction treatment providers need to be heard now more than ever as a group with specific needs and challenges. It is increasingly appreciated that during the crisis like Covid-19 and the war, providers are at risk for burnout despite (or perhaps because of) being flexible, creative, resilient in coping with challenges and finding new ways to help everyone but not receiving much support themselves. Healthcare is an essential societal system, and the providers were used to being altruistic and professionally proud to deal with the ongoing issues. Due to the existing challenges the nature of the system pushed providers to their limits daily. Providers burnout was noticed in healthcare systems worldwide after COVID shown by the shortage of nurses and front-line clinicians (6–9). Throughout COVID data came from multiple places that providers developed the biggest mental health impact in terms of anxiety and became the most affected group in comparison to other groups (10–16).

In sum, this article takes the readers back to the pre-Covid-19 Ukraine to find solutions to today’s challenges and to see what the learnings from the past can teach us in dealing with the aftermath of humanitarian crisis today. First, it describes the setting of addiction treatment in Ukraine, then presents findings from the original qualitative study of addiction treatment providers and interprets learnings from these findings from the contemporary vantage point.

## Setting

### The overview of OAT programs in Ukraine

To understand providers’ accounts in the Ukrainian context before the Covid-19 and the war stroke, we gathered information from policy documents of the Ukrainian Government, from experts at the Ukrainian Alliance for Public Health (i.e., Alliance), and other non-governmental organizations (NGO) reports.

#### Establishment of OAT programs in Ukraine

Ukraine is a LMIC with a population of approximately 42 million. At the time of data collection, it was estimated that there were 340,000 PWID, mostly of opioids, 4.5% of whom were enrolled in OAT programs throughout various regions of Ukraine. Yet, to curb HIV and HCV transmission, the World Health Organization (WHO) recommended that at least 20 percent of the estimated number of PWID receive OAT (17).

Opioid Agonist Therapy (OAT), in the form of buprenorphine tablet, was introduced as a pilot in 2003 by the Global Fund for HIV+ harm reduction clients (18, 19). Once it proved to be successful, the program was expanded countrywide in 2004. A pilot methadone program was started subsequently, and by 2008 MAT included both buprenorphine and methadone. By the end of 2021 only 10% to 15% of all OAT patients in Ukraine received buprenorphine as methadone is cheaper ($4 vs. $12 per patient per month) (20–22).

Historically OAT programs started in high priority areas such as Kiev, Dnipro, Odessa, and Mykolaiv where drug use and HIV were more prevalent to address the HIV-tuberculosis (TB) crisis. The first sites, funded by Alliance and The Clinton Foundation, were integrated into existing drug addiction clinics, TB clinics, and city AIDS centers. The Ministry of Health (MoH) regulations for OAT sites were based on experience of other countries, and some local knowledge from the Ukrainian stakeholders. The regulations were developed on evidence-based practices for addiction treatment and were carefully thought through and controlled throughout the country, with very careful measured implementation, and the measures of control were based on social dialogue among various stakeholders and some consensus that was built. The development was not fast and took its time and every step of the way was done thoughtfully and cautiously.

The first sites were located on the outskirts of cities where TB hospitals, AIDS centers, and addiction clinics were located. Eventually, new sites were opened throughout the cities (19). Since 2018, some OAT programs were integrated into primary care medical facilities.

According to communication with Alliance at the time of the study, there were approximately 947 narcologists across 211 OAT program sites and 11,385 patients (or 3.3% of the estimated population of PWID) were enrolled throughout Ukraine (23). Sites varied size-wise between 6 and 300 patients, and some had waiting lists while others had patient vacancies. The MoH (Center for Public Health) determined the number of treatment slots for each region.

The regulatory framework for OAT provision in Ukraine includes the federal (i.e., national) law #693 in effect since 1991. This law mandates that all patients receiving treatment for addiction are registered in the national drug user’s database. Studies showed this is the biggest disincentive to new patient OAT access as employers and governmental departments check this database before employing a person or issuing a driver’s license (3, 18). De-implementation of employment or driving restrictions for OAT patients were advocated, but changes were slow. The second regulatory element is the Order 200 passed in 2003 and revised in 2015 to allow take-home dosing to OAT patients who meet certain conditions.

## Methods

The senior author collected the data in 2017 and the first author conducted data analysis in 2018–19, as part of her dissertation project [unpublished]. As the crisis began to evolve, we returned to the analysis and reconsidered its findings for insights that can help to understand the processes in the addiction treatment system of Ukraine in response to the crisis, as they are currently evolving. Knowing the data collection was completely unbiased to the current crisis, upon re-examination, certain points appear to be very sharp given the light we can now shine on them.

## Participants

The data came from 24 qualitative (semi-structured) interviews conducted in February 2017. The interview participants included front line clinicians of OAT programs, doctors running OAT programs and senior clinical directors in equal proportions. Every effort was made to ensure the diversity of the sampled participants in terms of the mix of these seniority/roles in each region and across the regions. Five geographic regions were chosen based on their diverse location as well as OAT situation and randomized the providers from there. Recruitment was conducted through professional networks of OAT providers in Ukraine with whom we have been working with since 2015.

One way where a qualitative approach is most powerful is in a circumstance like this, where very little to nothing is known about the circumstances of the group in question (i.e., OAT providers in Ukraine) and formative work needs to be done to learn and *understand* their context-specific accounts and from these individual accounts, build a rigorous collective story, in which other OAT providers (in Ukraine but also possibly elsewhere) may recognize themselves and their experiences.

## Instrument

The purpose of the interview was to capture the experience of providers working at and running the OAT program in Ukraine. The interviews included conversations about what types of challenges they faced in running these programs and how they resolved or coped with these challenges. Importantly we received feedback on what aspects they were particularly satisfied with. During the interview process we kept a keen ear open for details on barriers and facilitators given the push to scale-up the program in the near future. The data came from 24 qualitative interviews conducted in February 2017 using an interview guide (Appendix B) with clinicians from the OST programs in Ukraine. Please see Table 1 (Appendix A) which outlines a list of participant attributes. As previously mentioned, in order to capture diversity, participants were ~ equally recruited from five different regions in Ukraine (so that about the same number of participants are from each region). Approximately 1/3 of participants were front-line clinicians in the OST programs (junior doctors, nurses). Approximately 1/3 of participants were doctors running the OST programs. The remaining ~1/3 of participants were senior doctors who held senior leadership positions in their respective hospitals/organizations (e.g., Head of the regional AIDS Center) and who oversaw the OST program as part of their job’s mandate. Every effort was made to ensure the diversity of the sampled participants in terms of the mix of these seniority/roles in each region and across the regions. Five geographic regions were chosen based on their diverse location as well as OST situation and randomized the providers from there.

Recruitment of participants was facilitated by the Ukrainian Alliance for Public Health, an organization that has strong networks and good relations with addiction treatment clinics across Ukraine. Each interview was 20–25 pages in length, lasted from 35 to 60 min and was conducted in person by the last author in the language of the participant’s choice – either Russian or Ukrainian. Interviews were transcribed verbatim and translated into English by qualified translators, the quality of the translations were verified by the second and the senior authors who are native speakers. Interviews were anonymized for protecting participants’ confidentiality; identifying information such as names of clinics or names of participants is not disclosed.

## Analysis

We utilized Dedoose (version 8.1) software (2018), a cross-platform app for analyzing qualitative and mixed methods research to manage the data. The first step in the analysis involved the task of reading and coding each excerpt into a coding tree where it can then be further analyzed to better understand the experiences of running an OAT program in Ukraine.

To develop the coding tree, parent codes were created. A parent or root code is a more general description of topics that are discussed in the interviews. Child codes were then added where necessary. A child code is a subordinate of the parent code and is a more specific sub-topic that is discussed within topics. For example, one of the parent codes is “barriers” and the child codes are “procurement,” “social barriers,” and “structural barriers.”

The process of creating the codes was inductive as all of the members of the team read a sample of the interviews. We then had two rounds of discussion to formulate the core codes for categorization and create the coding tree in Dedoose. Once the core of the coding tree was set, we focused on creating the subordinate or child codes, we then re-read the interviews and categorized each excerpt into the appropriate code on the tree. Some excerpts could be cross-coded into two categories depending on their topic of discussion. For example, if a patient was previously discharged for diversion and was then allowed to re-enter the program the excerpt would be coded under the parent code “*clinical challenges* (sub-code: *diversion*)” as well as the parent code “*OST programmatic structure* (sub-code: *administrative discharge*)” and parent code “*rules for access* (sub-code: *return to OST*)”. Using a coding scheme of 103 inductively developed categories we explored participants’ perceptions of the program. These categories were aggregated into 15 parent codes which in turn were aggregated into three broader interconnected themes.

## Results

Analysis of the data from the interviews with OAT providers revealed three themes on how they understand what shapes success of an OAT program: legal and economic underpinnings of OAT provision, program’s organizational structure, and professional identity of providers. Economic policies challenged quality of care by uncertainty in program funding, inadequate staffing, and challenges surrounding treatment scale-up. Programmatic structure challenged success by limiting patient capacity, and difficulties around inter-site collaboration that hindered coordination of care. Yet success was supported by a sense of professional pride in patient achievements, being recognized as an expert, and being included in decision-making.

Three major interconnected themes focused around:

1. **Economic uncertainty at the institutional level (leading to under-staffing)**

Providers were aware that economic sustainability of the program and its ability to endure overtime, provide jobs to clinicians, and medication to patients depended on macro policies of Ukrainian government or international institutions like the Global Fund. In the following excerpt, an OAT provider shared their perception of economic uncertainty for their program as the Global Fund was expected to offload greater responsibility for funding the programs to the Ukrainian government (and providers were unsure if the government was up to that challenge). Concerns included the supply of medication and insufficient clarity of policies for procuring it.

[Provider 13: *“This incompleteness of this part of chain to supply the patient with drugs and the same with the addiction inpatient care. Now, the Global Fund will end, where should we buy; what prices/agreement?…we do not have such experience”*].

Providers’ accounts stated OAT programs were understaffed, and already exceeded capacity causing reluctance to admit more patients without further resources (being paid more, being promoted, or hiring more staff). According to communication with Alliance, the average monthly salary of a front-line nurse and even a doctor was very modest (the equivalent of $200 USD for nurses and $400 USD for doctors).

The status quo before the crisis meant under-resourced as far as staffing is concerned. The provider commentary below describes shortage of staff to provide care to the large patient body.

[Provider 18: *“R: Well, talking about narcological hospital, the staff specifically consists of 198 persons, so it is not enough. We have 15,000 patients registered here but they include not only patients with drug addiction, but also alcohol addiction, about 2,400 patients have drug problems. We have 765 patients on substitution therapy in oblast at 13 sites, and we have 226 receiving Methadone, and 61 receiving Buprenorphine. This is the distribution; they have one doctor and four nurses on staff.”*]

The quote from the provider below points out that there may be gaps at the organizational level regarding provisions for compensation (extra compensation) to clinical staff that provide OAT in addition to their general work mandate. OAT providers recognize these gaps and feel concern, dissatisfaction, and anxiety.

[Provider 18: *“medical staff work at narcologist office. The substitution therapy created the extra workload for them, and they wanted to receive some financial support for that. If the manager of the health care facility at raion does not find the resources for extra money, then there is tension.”*]

The excerpt below talks about motivation that is both economic but also intrinsic, as clinicians love their job. Yet there is concern that lack of economic remuneration and unclear priorities within the program structure, excessive work burden, and burnout, lead to staff turnover.

[Provider 14: “*The workload is huge; no one released us from our principal work, now this work has to be principal, but here in my unit, the principal job is OAT, this cannot go one without another. They go together. Here we need a lot of motivation, and we of course have those who do not stay for long.”*]

The next provider quote echoes the same sentiment in their account, when asked.

[Provider 18: I*: “And do you have high turnover in personnel and patients?”* R*: “…the retention in the program indicators were not so good. But when we could arrange more stable personnel in the staff, the patient adherence became better.”*]

Meeting operational needs with adequate staffing has a direct impact on the functional framework of the program as well as on improved patient adherence. Consistency among staff provides job satisfaction to providers and increased stability and structure to patients, whose good outcomes in turn motivate clinicians to continue working.

1. **Structural capacity of the program**

Is bigger better? Does success equate to more patients and/or staff? Providers were sure of one thing: being over capacity was a recipe for failure. There has been continuing pressure by the Ministry of Health on providers to scale up their programs while not increasing their resources and their staff. In providers’ view, the size of the program was shaped by the programmatic and institutional level regarding how much medication was procured and how many staff positions the clinic could fill given its public funding. Thus, the program’s potential for growth determined which patients are prioritized if only scarce slots are available.

The following two quotes raise the issue of whether retention may be the absolute goal or whether, some patients were better to be let go, for the program as a whole? Providers struggled to find consensus between meeting general administrative rules and requirements and acting in the best interests of individual patients.

[Provider 4: *“We have had, and we keep getting patients who quit the therapy. The reasons why our patients quit the program is …mainly because they either die or get arrested.”*]

[Provider 10: *“There are no patients who drop out (unless they get ill); it’s about 1–2%. Mainly all patients who were enrolled stay in care for no less than six months; some of them have been here for years.”*]

In the pre-crisis status quo, a range of styles existed in which facilities and programs were run. Some were more rigid and took the guidelines literally while others were more relaxed and creative in interpreting the guidelines. Here are two examples of very different approaches.

[Provider 13: *‘This is one case when there was an attempt to remove Methadone from the site and we expelled the patient from the program and kept out for 6 months, then we wrote her the conditions for staying in the program and now, thank God, she comes at a certain time, strictly observing all the rules of staying on the site.”*]

[Provider 20: *“We have not done administrative discharge for as long as 5 years.”*]

The pre-crisis status quo included the realization that things were not perfect; there were grievances and wishes that things could be better. Some expressed concerns that without better resources it would be impossible to improve the quality of care; all the while the MAT programs were somehow persevering. Providers wish lists included keeping the workload of clinicians manageable by increasing the number of sites and clinicians so that they can provide care at the appropriate standard.

During the interviews, providers were asked to share what changes they would welcome to increase efficiency. Here is what providers put on their “wish-list.”

[Provider 11: *“increase a number of [OAT] sites to make sure that this type of support would be better accessible to anyone who wants to receive it. …there are still certain limitations of how many patients may be enrolled and there are still lines and waiting periods. Plus, it is very difficult to negotiate transition of patients when they travel during the summer…to make sure they are provided with medication at their travel destination. Our partners always respond by saying that they have too many such requests and they simply cannot accommodate all of them.”*]

Many of the wishes for what the providers would like to see happen differently, could address the challenges mentioned previously – e.g. diversity of sites can make it potentially easier to cater to diverse challenges of patients.

[Excerpt from interview 23: *“I would like to see the increase in the number of the sites because we have many patients that come to us from different places of Kyiv region because they do not have any sites of this kind closer to their hometowns. That’s why they come here. …there should be more sites like this out there as many of the patients say that these sites are their salvation.”*]

If scale-up were to occur, the division of labor and job description would continue to be a concern on the organizational level. The following quote expresses concern that providers will have less time to treat patients and suggests that staff scale-up should occur simultaneously with patient scale-up and should be implemented at the policy level.

[Excerpt interview 17: *“There is one rather unresolved issue related to this idea of project scale up. And the issue is this – am I supposed to, as a doctor, fill out detailed information for each patient or can this be done by someone else? Resolution of this simple issue would greatly help us to move on further in our work.”*]

The staffing was the function of the funding for the programs, which was decided by policies – either Global Fund or the Government had to clearly put aside a certain budget for the staff. This did not come from the local clinic/organization budget – at least, not at the time of the study. A common theme was that a successful program – in the staff’s mind – was one that gave them a continuous job. Thus, economic stability of the program was one such universal issue. This was associated with OAT providers’ expectations for whether their jobs and employment would endure, but it also was connected to the OAT providers’ wish to have the mark they felt they made on addiction treatment in Ukraine, to endure too. Accordingly, the third key theme is that of professional identity and pride.

1. **Labor of love: Clinicians’ professional pride (and individual level of authority)**

The findings present providers’ individual-level experiences, that embraced their feelings of professional pride and ways in which they experienced and wielded their decision-making authority in their work. There is a range of levels at which the success can be understood and experienced from the point of view of clinicians. It can be understood from the clinical outcomes of patients, and also can be understood by providers having professional pride and satisfaction in the quality of care they provide to patients, the reputation their program and leadership has in the region and throughout the country. Professional recognition for achieving successful patient outcomes was important to clinicians feeling effective in their fields.

[Provider 16: *“Well, who comes to our clinic, the doctors do not go anywhere because (name of head clinician) has created the conditions for work, you should understand, it is very important when you go to work and know that you will be heard, you will be understood, you will get help finally. That’s why the staff of doctors are very stable.”*]

Is a successful program one where the clinician is professionally successful? Clinicians want to feel recognized as experts in their field by peers from other clinics and regions. They appreciate being consulted and want to consult others.

[Excerpt interview 16: *“That’s why we collaborate very closely. If our patients need consultations, we start with our specialists. Yes, all doctors have the highest ranks, and they know peculiarities of the patients with addiction. It is our specialization.”*]

As the participant explained below, being recognized as a leader in their field was very valuable and important for them, especially as this recognition came from both domestic and international colleagues. From this point of view, a successful program was one that was recognized as such by global peers in the field of addiction treatment.

[Excerpt interview 16: *“I was the only one out of our specialists that received this certificate of leader in Ukraine. The American colleagues recognized the services of narcological clinic as the best and handed me this certificate. We are really the best.”*]

There are different kinds of clinicians (some frontline distributors such as narcologists and nurses where OAT is their only job), and then there are some senior clinicians who are like medical directors of large hospitals who have a portfolio of many departments to oversee, and this (inpatient) drug treatment program is only one of them. The excerpt below illustrates an account from a provider who was at a more senior level. He directed the inpatient drug program under the auspices of the hospital and was less connected therefore was less concerned than the frontline clinicians, as his job was not as affected as the site narcologists and nurses. To this participant, success in terms of his own profession, had a broader meaning, and stemmed from doing well in the management of the large healthcare organization, of which the OAT site was a part. His reference to “global” suggests that he emphasizes the importance of his role and of how well he performs it, for the overall performance of the large healthcare organization.

[Excerpt interview 22: *“The most inclusive definition of my responsibilities is making sure that the hospital remains functional and constantly developing. This includes the guidance related to global level policy, global level hospital management, and global personnel issues. I assign people for the positions of deputy chief physicians, for the positions of the department heads. I am also responsible for hiring physicians of this hospital. The head of HR may search the candidates, but every physician has to have an interview with me to be hired. I am directly responsible for implementation of the city program for HIV infection and also city policies development regarding HIV.”*]

Some clinicians were proud of their patient’s achievements (making family members happy) and others see it as an award for themselves (giving them more authority, having influence on decisions in the medical system).

Although take-away medication and prescription privileges are allowed state-wide under Order 200, the program is not offered at every site and is at the discretion of the narcologist. Currently, more than 1/3 of patients across the country receive some form of prescription/take home medication. The change in Order 200 in late 2015 that authorized prescription/take home medication was a game changer for Ukraine, and largely due to the work of ExMAT (3). There are some locations where the site narcologist will not allow these privileges and want every patient to come to the site every day. They will not dispense medication any other way. The large site at the Kiev City AIDS Center is among the sites that do not allow their patients to receive take-away dosing or prescription privileges. The narcologists and the administration of the medical facility at whose premises the OAT site is located decide on this for each individual site. The administration role is equivalent to that of a medical director in U.S. practice.

The three main criteria for new patient admission into an OAT program are registration into the federal law 693 database, having 2 failed attempts to stop using illicit drugs documented in the medical record, and proof of opioid addiction. Registry into the federal database is not a modifiable rule though there is a growing private market that provides long term “detox” using OAT without registering people. Some rules, however, are modifiable at the organizational level of individual OAT sites (at the discretion of the individual site narcologist). For example, some site narcologists will be flexible about the 2 documented failed attempts. Some will go so far as to keep the client in the inpatient clinic for 2 days and document it as 2 failed attempts.

Additionally, some site narcologists will relax the rules of proving opioid addiction by having the patient show up in the morning, perform all of the necessary tests and give the medication on the same day. In stark contrast however, some site narcologists will keep the client at an inpatient clinic for 6 days and observe their withdrawal symptoms to prove their true opioid addiction. Having two failed attempts and proving opioid addiction is a requirement of Order 200.

According to personal communication with the Alliance experts (February 27, 2019), there is a variation in application of discharge rules among the individual sites and it is determined at the narcologist’s discretion. Some sites do not administratively discharge patients therefore the only retention loss experienced (outside of patients’ voluntarily discontinuing treatment) is when a patient either dies or goes to jail. As an alternative to discharge, if a patient presents with a urine test that is positive for opioids, the narcologist will work with them to slightly increase their dosage to a comfortable level to deter them from using illicit drugs. Contrastingly, some sites will discharge a patient for a urine test that shows positive for cannabis. In sum, even though the system of OAT provision in Ukraine is heavily regulated at the national-level and is based on strict surveillance policy principles that must be observed at all times and in every place, there are also interpretations of organizational-level policies (allowed by Order 200) that may differ somewhat between individual organizations (i.e., OAT sites), where the narcologists have considerable power to “manage” their individual site and can adjust the rules of day-to-day business of OAT provision in accordance with the needs of their daily operations, if they choose to.

One example of this organizational-level policy is take-away dosing. Take-away dosing is when a patient receives a 10-day supply of medication to take home with them. Take-away dosing allows more freedom, less restriction, and more flexibility for the patient. This is a privilege that patients must earn in order to be eligible. To qualify for take-away doses a patient must be enrolled in the “everyday” program for at least 6 months and have a urine test history that is negative for opioids and sometimes also for other illicit drugs. The patient must be comfortable with their current dosing, not experiencing any withdrawal symptoms, have no administrative violations (i.e., being well-behaved and not violent or disrespectful toward staff). The induction begins with a trial period for one weekend, and then progresses to 3 days, then to a week, and finally a 10-day period. The site narcologist can revoke this privilege at any time as a result of a urine test positive for other drugs including cannabis and alcohol. Narcologists may also test the patient and ask them to come to the site when not scheduled and to bring their remaining medication, at which time they will perform a random pill check to test appropriate at-home dosing to confirm compliance and that no diversion of medication has taken place.

During the pre-crisis period OAT programs were regulated by policies and laws, providers had some discretion regarding the structure of their program and changes at their sites. Some narcologists used this discretion to benefit the patient by creating interpretative flexibility around rules of entry and retention such as not having to prove two previously failed attempts before entry, relaxing harsh rules for administrative discharge, and by allowing take-home dosing privileges (once qualified) so they did not have to come to the site every day. In contrast, some site narcologists used their discretion to strictly enforce the program rules. Providers’ decision-making authority determined who/how many clients the site had, and under what conditions these clients were kept. Thus, one example below suggested how providers preferred everyday attendance by clients, whereas the second example illustrates how a provider shaped a program that is more supportive of a certain kind of vulnerable client (i.e., pregnant women).

[Provider 11: I: *“Do you have a possibility to provide medication by prescription here?* R*: No, we do not. They only have this possibility at Demeyevka.* I: *Ok, and do you have possibility to provide patients with medication for a number of days or do they have to come every day?* R*: No. They have to come every day!”*]

[Provider 11*: “If a woman is pregnant, we accept her into the program without any lines, waiting periods and even without medical examination and tests. Such women are offered treatment from the very start.”*]

At times, site addiction doctors preferred to control all program’s activities and have frequent interactions with the patients to control them.

[Provider 4: *“We have been gradually expanding, very slowly. We can see our opportunities in expanding only provided that we issue the medications … Methadone among them… to the patients personally.”*]

The authority of individual clinicians was a process rather than a constant, and their criteria of what they thought a good working program was, may transform and change over time as expressed in the following excerpt.

[Provider 17: *“what they love the most is Demidrol. They can shoot up to 40 cc a day. I tried to prevent that particular predilection by providing* var*ious explanations and reasons on why that should not be done, but once there were only few patients left in my office with me and one of them said: “You know what? I simply cannot live without this stuff. And this is how I feel all the time no matter what dosage of methadone I receive.” Plus, at some point I had a very informative conversation with [Name of foreign expert] on this topic and little by little I changed my point of view concerning this matter. It is not that bad after all, I thought to myself. And there is no reason to kick a patient out of the program like we used to do it before whenever we learned about patients doing something of that kind.”*]

Over the years there have been policy changes, promoted by efforts from the providers themselves. Implementation of the Order # 863 was a milestone victory for Ukrainian OAT providers who could now offer different forms of prescription services for those who qualified.

[Provider 17: *“Everything is regulated by Orders of MOH; our main Order is the Order #200 with its multitude of appendixes. Then… we got this new Order #863, which regulates prescription forms…And it was then that we were able to get both – free and charge prescriptions going.”*]

The introduction of liquid methadone – a policy decision – was expected to reduce diversion, potentially resulting in fewer discharge occurrences. It would also reduce clinicians’ burden from policing the patients to confirm they have swallowed the medication (and not hidden or “cheeked”). Liquid methadone is easier to inventory (because of software) and it tastes better (24).

[Provider 17: *“Because, I am personally not sure at all that all our patients take their medication orally the way they are supposed to; some of them actually inject it; They begin to inject more and more and may easily achieve the dosage when overdose is likely to happen. The only solution of this problem is introduction of the liquid form of methadone. It is a must. That is what they did in the U.S., and they did it for these very reasons.”*]

Reducing the opportunity for diversion could positively impact MMT adherence thus creating more “good patients” providers want to see at their site. Good patients will help with the longevity of the program to create a successful image so it will continue to be funded.

[Provider 17: *Some of our patients continue to inject themselves with more methadone to achieve a desired condition. Methadone has become a drug that is much easier to get hold of these days. They have learned to synthesize methadone here just as fine as everywhere else. … I think that things would have become a lot easier if we had a liquid form of methadone as they do in America.”*]

Professional recognition for achieving successful patient outcomes was important to clinicians feeling effective in their fields.

[Provider 16: *“Well, who comes to our clinic, the doctors do not go anywhere because (name of head clinician) has created the conditions for work, you should understand, it is very important when you go to work and know that you will be heard, you will be understood, you will get help finally. That’s why the staff of doctors are very stable.”*]

As expressed by provider 16, the need to be heard is substantial. It seems that clinicians feel they are only heard, and their views are taken seriously by their peers. This echoes extant literature’s findings from developed countries, except that their providers were heard about their satisfaction or dissatisfaction across a wider range of domains, and in our study, it seems that providers were at best hearing one another (but overlooked by policymakers).

While in some ways our findings echo results from developed countries (in the general point that economic sustainability is important), our participants were very vulnerable vis-a-vis the authorities that provided funding. They were reticent in expressing dissatisfaction with the existing policies lest it compromised resources for their facility like medication supply and salaries funding. It seems there was more anxiety present than there was dissatisfaction. Our participants expressed feeling more acted-upon than heard.

Since when the participants shared their perspectives, some changes occurred, most notably, the Covid-19 pandemic and the Russian invasion of Ukraine. While at the time of data collection, OAT providers only began to offer several days’ prescription of OAT, during Covid-19 lockdown most OAT sites started giving 10-days-worth take-home medication or prescription form of OAT, to all patients to whom it was clinically safe to do so. Thus, the need to treat vulnerable PWIDs during Covid-19 lockdown helped creatively address challenges that have been long undermining programs’ success at policy or organizational level, such as streamlining rules of entry and retention, and expanding take-home dosing privileges, items that had been on OAT providers’ wish-lists for a while.

## Discussion

In September 2023 as we were finalizing this article, we conducted consultations with five addiction treatment providers in different regions of Ukraine (Kyiv, Donetsk, Zaporizhzhia, Kherson, and Zhytomyr) that have been close to the frontline. We did that to review our findings from data collected in the pre-crisis era and consider lessons that can help better understand the present-day challenges and develop more robust solutions. Questions we discussed with clinicians spoke to the three themes in our findings, including access to resources in humanitarian setting, changes of patient population of their programs, and what factors supported them to continue doing their job despite the non-ending risks, including of physical destruction.

These consultations reinforced that chronic and systemic problems of the healthcare system in Ukraine predate the crisis. Resources that healthcare workers, facilities, and the healthcare system command pre-crisis may be the key factor in predicting how successfully the crisis may be withstood. Noting the abrupt changes that occurred places emphasis on the importance of the providers unmet wish-list items. These perceived pre-crisis deficits are important because the healthcare system was not given warning or time to compensate for them, given the suddenness of the pandemic followed by the war. The crisis placed further demands on providers against fewer resources while chronic system’s problems remained.

### Resilience

For decades, while adhering to the Ministry of Health rigid rules narcologists have practiced creativity to withstand challenges like economic uncertainty, shortages of funding and of medication supply, and understaffing. Yet, participants valued their work, the sentiment rooted in their professional identity where being a narcologist is a calling. This pride in what they do despite the difficulties, is an ethic that helps participants to adapt to challenges, demonstrating resilience.

Our findings revealed the pre-crisis “Normal” or business as usual, i.e., the experiences of Ukrainian addiction treatment providers to work with limited and insecure resources, be creative to overcome hurdles, and seek contentment through work recognized by peers and patients as worthwhile. The resilience of an individual clinician or the healthcare system depends on how much pressure or stress it can withstand without collapsing, and for how long. Covid-19 and other crises have raised the question about the limits and costs of resilience, e.g., in terms of negative physical and mental health consequences for the healthcare workers themselves. According to gyeman-Manu et al. (25) and Ghebreyesus (26) chronic underinvestment in the healthcare workforce coupled with an imbalance between available staff and patients’ demand has created persistent labour shortages, exacerbated by Covid-19 pandemic and its aftermath when health workers had to operate beyond human capacity for extended time. One can argue that in Ukraine, operating at or even beyond human capacity pre-dates the crisis.

This brings two important questions. The first is whether experiences of operating with few resources for extended time prior to crisis may increase tenacity of individual providers and of the overall addiction treatment system during the subsequent crisis or conversely make them more vulnerable. The second is the long-term costs of resilience for individual clinicians (e.g., in terms of stress-related chronic health conditions later in life) and for the healthcare system. Answering these questions would require longitudinal research in Ukraine and is relevant across other global regions including the US, and as such, must be approached systematically. To improve population health, advance socioeconomic progress and promote human rights the WHO (27) suggested previously that healthcare workers be considered a human capital investment as independent investments in health will not effectively resolve workforce shortages. Perspectives of Ukrainian addiction treatment providers prior to crisis echo albeit scant research findings from other regions, suggesting they may be potentially affected by crises in ways similar or exceeding Ukraine. The humanitarian crisis that Ukraine is enduring emphasizes the need for better preparedness of the healthcare system to disasters, including paying more attention to healthcare workers’ perspectives to reduce resources insecurity in healthcare. This insecurity may have a gendered component, as men including clinicians are drafted during an armed conflict, leaving female health and care workers to carry out the clinical operations. This may present additional challenges as women are underrepresented in decision making and face a 24% gender pay gap according to WHO (28).

According to our findings from OAT providers, challenges that existed before the poli-crisis included economic uncertainty, lack of resources, insufficient structural capacity, understaffing, rigid rules on take-home doses due to risk of diversion and accessing distant site locations which created risk of retention, patient loss, and continuity of care. The precariousness of the economic foundation of the OAT programs was a common concern among clinicians. The long-term sustainability and security of methadone supply was a big concern, alongside economic stability of the program including sufficient funds for staff salaries in the context of uncertainty about the sources and availability of public funds, supply of medication and policies for procuring it.

### Theme 1: economic uncertainty at the institutional level (leading to under-staffing)

Like substitution treatment research in economically developed countries (29), studying Ukrainian clinicians’ perspectives before the crisis suggested that ensuring quality of care when scaling up OAT is contingent on sufficiency of resources including medication supply and staffing. Meeting operational needs with adequate staffing has a direct impact on the functional framework of the program as well as on improved patient adherence. Yet, the Ministry of Health policies in the period before the crisis focused on expanding the patient population without creating more healthcare jobs, reducing the number of small clinics, and optimizing, i.e., increasing providers’ patient loads, while simultaneously cutting some junior positions like nurses to reduce costs. In the addiction treatment providers’ experience, such policies risked undermining the accessibility of OAT sites for patients. While smaller sites, especially in rural areas, may not have been viable economically to stay open, amalgamating sites into larger facilities could result in both clinicians and patients traveling long-distance daily. Previous studies found higher drop-out rates associated with greater distance between patient homes and treatment sites (30, 31). During the crisis greater familiarity of clinicians with patients’ circumstances and a trusting relationship, that could be possible if caseloads allowed time for a more holistic approach, proved invaluable for the successful provision of medication, including wider use of take-home doses on a case-by-case basis. This flexibility may be encouraged by structural interventions (32) that facilitate more patient-centeredness without increasing providers’ caseloads.

Economic uncertainty that was a fundamental characteristic of OAT providers’ experiences pre-crisis, intensified since the Russian invasion. OAT providers were uncertain if the medication supply would be replenished or depleted (33, 34), but our findings indicate that they had to ration their inventory of medication already before the crisis during the “Old Normal”. Likewise, the war made staff shortages more acute, but they predated the crisis, especially among the junior frontline staff like nurses, who, unlike more senior specialists, could not perform their work via telehealth but needed to be physically present on premises and thus at high risk if the facility was hit and destroyed.

Remarkably, all the clinicians who shared their war-time testimonies with us as we were interpreting the findings in the current context, had worked in the profession for over 15 years, thus having in-depth knowledge of the pre-crisis context. While providers demonstrated considerable ingenuity and congeniality during the war proving Plato’s quote that “necessity is the mother of invention,” their insights underscore the need for disaster management planning and preparedness at the healthcare system level. While Ukrainian addiction treatment providers’ dedication to care for patients in the most volatile setting is commendable, the lesson from their experience is that improvising may not be the sufficient response, and careful planning of policies and allocation of sufficient resources pre-crisis is needed at the Ministry of Health level.

### Theme 2: structural capacity of the program

Our interpretation of the addiction treatment’s providers hesitation to expand their programs to admit more patients citing capacity restrictions (that echoes findings from developed countries) is that the “invisible hand of the market” prior to Covid-19 and the war, tightly squeezed LMIC clinicians by the throat. The OAT providers’ experiences during the pre-crisis period suggested that flexibility and creativity to better meet patients’ needs was healthcare workers’ core value. Yet, their efforts were undermined by insecurity of funding as OAT providers experienced pressures to expand their patient population without additional resources or new staff positions.

As control and surveillance over addiction treatment patients require significant resources from OAT programs’ staff (35), this model, common and feasible pre-crisis, had to transform during the Covid-19 pandemic and particularly since the Russian invasion. Thus, transferring stable patients to a 10-day (and occasionally to a 30-day) take-home methadone regimen during Covid-19 crisis, and using this practice after the Russian invasion, not only minimized patients’ exposure to risk but also allowed continuity of care despite imminent staff shortages due to illness and war challenges including internal displacement. Thus, trusting relationships between providers and patients nurtured pre-crisis facilitated maintenance of the programs’ capacity during the crisis when resources to support the program structures were in short supply. Knowing one another proved to be vital for major solutions during the crisis, as these networks of trust were fundamental for making take-home doses successful during the crisis, and later for getting medications to people who evacuated (33).

After the war crisis hit and one-third of the Ukrainian population was displaced, challenges quickly emerged, and OAT providers had to come up with creative approaches to alternate treatment practices in order to address disruption in medication service delivery. Patients who were already authorized for take-home doses were increased from 10 days to 30 days however patients who were required to present at treatment centers for their medication on a daily basis were at immediate risk for discontinuation. Furthermore, potential out-of-treatment patients whose illicit drug supplies were also interrupted and now seeking OAT were at risk for rapid withdrawal or suicide.

The Network for Improvement of Addiction Treatment (NIATx) model for behavioral health (36), originally introduced in 2014 to aid in the scale-up of OAT, is a bundle of implementation tools that promote the application of exercises to address key problems through coaching strategies and motivational lectures to manage growth change projects for guidance toward improved health policy changes.

According to Altice et al. (33), U.S. NIATx experts held weekly coaching calls with Ukrainian narcologists and the Public Health Centre which helped to guide OAT providers to quickly transition to appropriate treatment practices as the war intensified. NIATx coaches worked with the Public Health Centre to provide guidance to specifically address proper dosing and re-enrollment (37). Altice states that 858 (5%) of the 17,232 patients were lost in the first month along with the closure of 16 (6%) of the 277 OAT sites. Altice reports that1month later the OAT program increased by 6.9% due to re-enrollment of 1,136 patients and 6 months later reported an 18.1% increase which included new patients as well as internally displaced patients (33).

As OAT providers, the Public Health Centre, and NIATx coaches collaborated to strengthen the OAT delivery in Ukraine, it became clear that the providers professional pride coupled with the support of these experts became the cornerstone of the success of the OAT program in Ukraine. NIATx continues to guide and empower OAT providers to address emerging problems using evidence-based practices and to create novel strategies for preparedness practices to move forward to further respond to challenges that create barriers to OAT treatment during times of crisis or disruption. It is hopeful that this Shared Decision-Making approach will become the foundation when policymakers reconvene to restructure the program post-war.

Since the war, the need to operate with insufficient resources increased yet became in some sense more palatable to clinicians who felt they were thus expressing solidarity with their countrymen and contributing to a larger public good. As reflected in recent literature (1), Ministry of Health relaxed its rigid rules on take-home doses, allowing Ukrainian addiction treatment providers greater flexibility in providing care to patients, allowing expanded take home dosing (from a 10- to a 30-day supply) in order to continue care and minimize loss of patients. Risk of diversion remained. Clinicians also addressed the need for their patients to access distant site locations during the war, following abrupt interruption of medication and service delivery after reduced access to local pharmacies and closure of 16 OAT program sites in first month. They managed it by relocating to the Western region and communicated via Telegram-SMS to patients and their network of OAT providers (1). Yet, it signified the increased risk of patient loss/retention and continuity of care.

### Theme 3: professional pride

The pre-crisis functioning of OAT programs in Ukraine was a system where the understaffed clinics followed the rules of a top-down structure (5) where the providers had little authority, minimal salaries but ample professional pride to continue the mission. Findings point to systemic barriers pre-crisis however participants were proud of strategies they had developed to overcome the various deficiencies and shortcomings. Despite being over worked, understaffed, and underpaid the sense of identity fueled their continuous strive to do more with less. Yet, although professional pride is a noble, altruistic characteristic, its downside may be the burnout when healthcare workers are forced to operate beyond human capacity for extended times.

Recent research demonstrates (7, 8, 38, 39) that clinicians are expected to indefinitely provide an essential service under any circumstances despite personal risks. At 20 months since the Russian invasion of Ukraine, it appears that the most significant resource on which healthcare workers rely the most is their professional identity or pride, in the most positive sense. When the physical infrastructure crumbled (i.e., missile strikes destroyed clinic buildings) and the administrative rules that govern at the organizational level also crumbled as many processes became logistically impossible, professional pride – the intrinsic motivation to continue doing their work despite all odds out of genuine commitment to their patients – remained intact. Knowing how diligently addiction care providers had always worked with limited resources pre-crisis, being guided by their professional duty, helps better understand how during the crisis providers continue to endure ever increasing hardship without giving up. Yet, working beyond human capacity may come at great personal cost, and anecdotal evidence during the crisis shared in recent consultations by our informants from the addiction care in Ukraine suggests that overtime, medical staff may experience negative health outcomes despite their heroic tenacity, with some clinicians physically collapsing at workplace, yet others suffering from burnout. Alongside our own studies during the pandemic and during the subsequent war to unpack responses in addiction care (5), research suggests possible ways to assist and support providers during the crisis (1, 15, 40, 41) and maintain their professional well-being by adopting educational interventions through communication platforms like the Network for Improvement of Addiction Treatment (NIATx) model for behavioral health (36). According to Ivasiy et al. (1), participating in collaborative learning interventions with NIATx coaches, helps to alleviate healthcare workers’ isolation during the crisis (1, 37).

But as the humanitarian crisis in Ukraine continues, longitudinal research needs to focus on addiction care staff and examine burnout, resilience, and negative and positive coping strategies among frontline clinicians. While this has never been done before in Ukraine or elsewhere, our group has developed a proposal and been fortunate to receive funding to conduct a pilot study among 100 addiction care clinicians in Ukraine. Among other things, we will explore protective and risk factors associated with professional pride over time, e.g., correlations with healthcare workers’ burnout and resilience, but also their mental and physical health outcomes. Furthermore, given the emphasis that pre-crisis clinicians placed on support and recognition from peers within the professional community, in the forthcoming pilot study we will include questions on staff participation in professional development activities during the war (online and/or in-person) e.g., seminars, workshops, known to reduce burnout. We will also explore staff and clinical leaders’ perspectives on how to bolster both organizational and individual resilience and alleviate burnout, and what they do during the humanitarian crisis to that effect, and whether it is working well and may become candidates for scaling up, or not working in the humanitarian context setting and may need to be de-implemented. We will pay particular attention to whether and how during the crisis mitigating contingencies and working in constant overload mode may become normalized for healthcare providers, i.e., taken for granted. The study will also offer insights into the feasibility of co-producing approaches to alleviate staff burnout with frontline providers (and not only senior clinical staff), giving a voice and engaging them in identifying best practices in their facilities.

In sum, our consultations with addiction treatment providers during the war helped better understand the implications of findings from the pre-crisis study in the current humanitarian context. Reviewing healthcare workers’ experiences of coping with the various challenges and the strategies they already used pre-crisis reveals the roots of challenges that pre-date humanitarian circumstances and responses that may also be associated with increased risk overtime. While mental health issues and burnout are escalating among staff during the war, the ongoing healthcare reform to optimize accessibility, quality, and efficiency of healthcare services (42) places further uncertainties on funding for addiction care and current staff’s continuous employment given the impetus to shift some addiction care from specialized to primary care facilities with no clear pathways for staff re-employment. Considering the impact of Covid-19 and humanitarian crises, Dr. Hans Henri P. Kluge, WHO Regional Director for Europe (43) stated that healthcare systems will collapse without proper support if healthcare workers continue facing demands beyond human capacity (25, 26). Since the Russian invasion, experts feared that the Ukrainian health system may fail, destroying the hard-earned successes in high-priority areas of addiction care (33, 44). This also endangers the national and global efforts to combat the spread of HIV, as recent reports from Ukraine indicate an increase of nearly 6% in HIV diagnoses between January 2022 and June 2023, while individuals enrolled on antiretroviral therapy fell by almost 8% (45). While clinicians in addiction treatment in Ukraine maintain the continuity of OAT programs thanks to their deeply rooted professional identity and unique organizational culture despite the destruction and plummeting resources, assistance and respite is urgently needed – and it is not yet too late to provide it. This question also matters to the US, given healthcare staff shortages and a high rate of burnout (46, 47) during Covid-19 particularly among nurses (48–51), yet the government provided little response (52, 53), leaning into traditional expectations of healthcare staff sacrifice (54). The key learning from our pre-crisis research suggests that in low and middle-income countries like Ukraine, humanitarian crises strain already limited resources, leaving essential healthcare workers overlooked (5, 55). By shining a light on these essential addiction care providers’ perpetual and pre-crisis concerns, we hope to bring the trend of overlooking healthcare workers’ well-being to an end.

### Solutions/call for action plan post-crisis

Economic uncertainties and structural challenges are the chronic diseases of the Ukrainian health care system; they predate the crisis and will likely extend post-crisis therefore we are proposing a long-term wish-list to help bring about a new normality after the humanitarian conditions improve. Despite any challenges, some things remain relevant, including the increased need for OAT, healthcare workers’ professional pride and commitment to providing addiction services, and the hopefulness of collaborative approaches using coaching, learning, and support through Shared Decision Making. Thus, we identified some potential strategies toward a solution:

1. Economic uncertainties:
  - Indexing salaries would not only (partially) provide cost-of-living adjustments during the crisis but would help frontline clinicians feel valued.
  - The creation of a job bank for re-employment assistance could help displaced healthcare workers who lost jobs due to facility destruction.
2. Structural capacity
  - Reducing staff during ongoing humanitarian crisis (e.g., due to services restructuring) is unadvisable as it may be associated with increased anxiety and burnout of healthcare providers.
3. Professional identity:
  - Offering professional development opportunities may be a way to provide respite and offer motivational social support through communication platforms in an effort to sharpen skills, increase confidence and reduce burnout (15, 37, 40, 41).

Ukrainian OAT providers as shown by findings from our pre-crisis study, belong to a professional community that places high value on the success of their treatment programs. The OAT providers commitment to maintain these programs is an example of what Durkheim called organic solidarity, where cohesion develops from mutual support, overall conformity, and a strong sense of professional identity. As the findings suggest, while OAT programs in Ukraine were regulated and need to function to code, there was growing room for flexibility to improve quality of care. This resembles studies from other global regions (56–58) that highlighted the value of a professional community of OAT providers to advance topics like treatment goals and combination therapies. But in trying times, the supportive and protective role of a professional community can attain new levels (especially in the deficit of most other resources and supports).

Professional pride as a reaction to economic uncertainty and structural challenges places emphasis on commitment as OAT providers bring dignity and grace and they respect each other as a community assured by knowing what they do matters. During the Covid-19 pandemic this community was very difficult to maintain due to lockdown and then during the war it became even more complicated as it was difficult to communicate in-person and dangerous to move about the country. Although the ties holding the professional community together are fragile and resource vulnerable (no electricity meant no Zoom) OAT providers find ways to engage with professional development programs if they are only offered (1, 33, 34, 37, 59–61). This tenacity is inspiring and suggests that it can still be saved and rebuilt. Experience is the best teacher; let us learn from this experience. The Ukrainian proverb *“a hungry wolf is stronger than a satisfied dog”* means that it is better to be hungry for progress than to be satisfied with being stagnant.

1. Government can support healthcare providers by inviting dialogue and listening to the providers wish-list requests for serious consideration during the rebuild. Paying serious attention to healthcare providers’ well-being is imperative as burnout of healthcare providers during crisis is a growing concern that can have a global impact (6, 7, 9, 16). Importantly, the co-production approach common while developing successful interventions for service users must be also taken on board concerning interventions and policies directed at healthcare workers. Trust them to be their own saviors because they know what works and what does not through their lived experience. In post-humanitarian context, including addiction treatment providers’ vast experience into the development of treatment systems given the dearth of resources may prevent a cascade of negative implications within the primary care health care system, the Criminal Justice system, school systems and society in general.
2. Foster Shared Decision Making: This concept was mostly used in a narrow sense as it has historically meant to bring patients into the conversation when talking about the solution of their own problems (62–65). We suggest exploring the implementation of this technique in relation to providers and their dealings with policy makers who decide the future of SUD care in Ukraine (55). OAT providers have previously had little influence and their voices were dismissed. The Ukrainian OAT providers are (re-)writing the rulebook and other countries are paying attention wondering how they are managing this crisis a year (plus) into the war. Here, SDM between providers and policy makers could help to carve in roads as a way forward postwar. Thus, as part of our pilot study of addiction treatment clinicians in Ukraine, we will conduct a workshop with governmental officials from the Ukrainian Ministry of Health and frontline addiction treatment staff to discuss local adaptations to support the staff and continue providing addiction treatment during and after the humanitarian crisis. We will discuss the costs/sacrifices of sustaining addiction care provision in terms of clinicians’ own health, their organization’s staffing and patient care provision, economic remuneration, and other areas, from the perspectives of frontline staff. We will ask about any strategies and programs set up in their clinic since the Russian invasion to support staff (paying particular attention to professional development opportunities which we know from the literature may alleviate stress and prevent burnout including in humanitarian settings). The focus on lived experiences of frontline staff is of paramount importance to enabling bottom-up approaches to future interventions, and shape recommendations for individual staff, clinical directors, as well as for the government / policy making. As Shared Decision Making (SDM) and co-production is widely employed in work with patient populations, we will synthesize, in collaboration with the frontline addiction treatment clinicians, the insights from the workshop to form the basis for a report of recommendations that may ultimately improve patient outcomes, protecting addiction care in Ukraine from collapse. For example, inviting clinicians’ input on how to optimize roles of existing staff to scale-up OAT provision by expanding the take-home OAT on a case-by-case basis may alleviate clinicians’ perceptions of being overworked and underpaid (66). In the emergence of the new normal, communication between OAT clinicians and policy makers would be key to improve quality of care, improve providers’ job satisfaction and retention, and improve patient outcomes, by institutionalizing best practices within the clinicians’ professional community. Co-production by addiction providers could be the needle that weaves the common threads of wisdom to create a tighter textile of policies.

Our participants in the pre-crisis study, as well as addiction treatment providers with whom we consulted during the war to better understand the study’s current implications, entrusted us with their stories in the hopes we would understand. Not being spoiled by attention from either researchers or policy makers, addiction care providers in pre-crisis Ukraine had shared with us what they saw as the system’s shortcomings, and we ought to shed light on it after all these years. It is time that healthcare workers’ input is recognized, and their well-being is supported, and it is always better late than never.

## Data availability statement

The raw data supporting the conclusions of this article will be made available by the authors, without undue reservation.

## Ethics statement

Ethical review and approval was not required for the study on human participants in accordance with the local legislation and institutional requirements. Written informed consent from the participants was not required to participate in this study in accordance with the national legislation and the institutional requirements.

## Author contributions

PD: Data curation, Formal analysis, Methodology, Writing – original draft, Writing – review & editing. AM: Data curation, Writing – review & editing. TF: Data curation, Writing – review & editing. JR: Conceptualization, Data curation, Funding acquisition, Investigation, Methodology, Supervision, Writing – review & editing.
