# Supplementary material for: Re-examining provider perceptions of best pre-war practices: what elements can help opiate agonist therapy programs in Ukraine successfully survive the crisis?
Source: Front Public Health. 2024 Jan 4;11:1259488. doi: 10.3389/fpubh.2023.1259488 (PMC10794633; doi:10.3389/fpubh.2023.1259488)
Supplement: Supplementary file 1 [file Data_Sheet_1.docx]

**APPENDIX A: Participants**

| **TABLE 1: Attributes of Participants** | | | |
| --- | --- | --- | --- |
| **REGION** | **GENDER** | **ROLE** | **AFFILIATION** |
| Lviv | F (5) | 3 Narcologists  1 Head Narcologist  1 Chief Doctor | 3 Regional AIDS Center (RAC)  1 Head of Region  1 Drug Addiction Center Dispensary (DACD) |
| Odessa | M (2)  F (3) | 2 Narcologists  1 head Narcologist  2 Chief Doctors | 2 RAC  1 Head of Region  2 TB Clinic |
| Kyiv | M (2)  F (3) | 3 Narcologists  1 Chief Doctor  1 Nurse | 3 City Narcologic Clinic (Sociotherapy)  2 City AIDS Center |
| Kriviy Rig | M (1)  F (1) | 1 Narcologist  1 Chief Doctor | 2 City Mental Clinic |
| Dnipro | M (1)  F (2) | 1 Narcologist  1 Head Narcologist  1 Chief Doctor | 1 City Infectious Disease Clinic  1 Head of Region and DACD  1 City Narcologic Dispensary |
| Mykolayiv | M (5) | 2 Narcologists  1 Head Narcologist  2 Chief Doctors | 2 Central District Hospital  1 Head of Region and DACD  1 DAC  1 TB Clinic |

**APPENDIX B: Interview Guide**

**Draft interview guide for key personnel**

**Head Narcologists of the Region / OST site administrators / clinicians, doctors, nurses**

This Interview Guide is a DRAFT. In line with the qualitative interview approach, the Guide is a flexible tool to direct the conversation with the participants and to elicit their stories regarding the key domains listed below. The interviewers will be using “active listening” techniques and will have discretion to decide the order in which they may bring up domains, ask questions, and use further probes during the interview with each participant, to gather the richest data possible. The interviewers may also use additional questions and/or probes not listed in the Guide if the participant brings up relevant issues that may be worthwhile to explore further.

Preamble: **Purpose: Establishing initial rapport**

{Meet and greet the participant, ask how they are doing, ensure they understand the interview process and reinforce that their story will be valued and will be taken seriously and with respect}

“We are hoping to learn more about what the staff who work in the OST provision in Ukraine think about addiction, methadone maintenance therapy, and recovery, and the experiences they had with these issues in course of their work. We would also like to know your thoughts about how and why some people may initiate or discontinue OST, about the clinicians and patients’ relationships, about attitudes towards methadone, and about support for people living with substance abuse disorder. Your input will be very helpful in designing an improved program for PWID to encourage the use of harm reduction services, improve their quality of life, reduce stigma and social isolation, and reduce the HIV risk.

I’ll be asking some questions and recording our conversation. I am eager to hear and to learn from you and your experiences. I am not here to look for right or wrong, and it is your opinion that is of value. We may be asking you some questions that may appear sensitive to you. While we would greatly value a complete and thorough response, we would not want to put you into any situation that may put you in jeopardy from any other employees, authorities, and /or patients. In situations where this may seem uncomfortable, there are a number of ways in which you may respond. This may include speaking abstractly about events or activities that may be related to others and perhaps not to yourself, as well as making sure that you do not provide a full name that might completely identify another patient and/or staff member. If there is a question you do not wish to answer, please kindly advise; however I ask you to be as open as you can and say what you think. Everything you share today will be kept confidential and only used in anonymized form for research purposes; it will not be shared with the health department, narcology department, or any of your colleagues. How does this sound? Do you have any questions before we begin? {Interviewer clarifies any questions that may arise}. I am going to start the recording now.”{*Start recording*}

1. **Work environment and culture**

I would like to start by asking you a few questions about the work you do. Could you describe your work to me?

- *What is your role and main responsibilities?*
- *How long have you been in your current role?*
- *What about your job is the most satisfying or personally fulfilling?*
- *What is especially difficult about your work?*

**II. OST clients**

**1. Who are your clients? What challenges do they face on and off the site?**

- *In your view, what are the most common reasons that people who inject drugs start OST*?
- *Could you please describe how a person becomes an OST patient at your site?*
- *How much time is needed for a person to become an OST patient and to start receiving methadone? Do you have a waiting list?*
- *How is the treatment plan chosen and what does it include?*
- *Could you describe a patient’s first visit to your site? What happens in the first week of therapy?*

**2. Client retention**

- *In your view, what challenges and risks do your clients face in daily life?*
- *What do you think can prevent some potential clients from starting OST?*
- *What concerns do patients voice about OST?*
- *Can you tell me some reasons clients leave OST programs? Or stop taking MMT?*
- *In your opinion what can help a client continue OST treatment? (Probe: adjusting dosage? Allowing take home doses? Providing prescription?)*
- *Do you have a way to keep in contact with patients who alter their treatment plan? How do you do it?*
- *In your experience, what could lead a patient to be administratively discharged? How often could it happen on your site? Could you tell me about any recent examples?*
- *Can a patient return to OST after being administratively discharged? How would they go about it?*
- *Do OST patients sometimes voluntarily discontinue taking methadone? How does it happen? In your view, why do they do it?*

**3. Attitudes towards OST and the meaning of recovery**

- *Given your experience providing OST services in your region, do you think that OST is good for patients? Does it help people overcome addiction?*
- *In your view, what do your patients think about methadone? How about their families? What do other providers/administrators think about OST? What is the attitude towards OST in the society in general?*
- *In your view, what is recovery, when we are talking about PWID who have got on OST?*
- *How do your patients think about recovery? What does it mean to them?*
- *Do OST patients sometime continue using street drugs? If yes, in your opinion, why do they do that?*

**III. Integrated Care**

- *What do you think about a possibility to provide OST in a primary care facility (outpatient clinic)?*
- *In your view, for what patients such integrated care treatment can work well?*
- *What may need to be done to transfer these patients to OST treatment in primary care facilities?*
- *How will doctors and nurses treat such patients?*
- *Do you think the quality of care will be better or worse than in specialized OST sites?*
- *Would clients like receiving OST in primary care facilities?*
- *What do you think about clients paying for OST treatment?*
- *What are your thoughts about clients purchasing OST from a pharmacy?*
- What may need to be done to ensure high quality of OST services in primary care facilities?

**IV. Environment /Macro factors**

- *In your view, what factors in your region make the work of OST site easier / more difficult? What affects attracting / retaining OST clients?*
- *Is there some interference of police in your job? If yes, what kind?*
- *Do you collaborate with other healthcare organizations / other OST sites? How does collaboration happen?*
- *In your view, how many PWID could you treat with OST? What may be needed to expand the OST program at your site?*
- *I heard that the Ukrainian Center for Disease Control now has the mandate to increase the number of people who inject drugs on OST to 14,000 based on Global Fund funding.*
  - *What would need to be done to accomplish this?*
  - *If you could change your OST program, what would you put in place to improve treatment?*
  - *What would need to be done in order to make the changes you suggest?*
  - *What gets in the way of making these changes?*

Thank you very much for your time. Are there any questions you would like to ask of me at this point?
